# Theoretical Confirmation of the Quinone Methide Hypothesis for the Condensation Reactions in Phenol-Formaldehyde Resin Synthesis

**DOI:** 10.3390/polym9020045

**Published:** 2017-01-29

**Authors:** Taohong Li, Ming Cao, Jiankun Liang, Xiaoguang Xie, Guanben Du

**Affiliations:** 1 The Yunnan Province Key Lab of Wood Adhesives and Glued Products, Southwest Forestry University, Kunming 650224, China; caominghappy@swfu.edu.cn (M.C.); liangjiankun@swfu.edu.cn (J.L.); 2 Department of Chemistry, Yunnan University, Kunming 650091, China; xgxie@ynu.edu.cn

**Keywords:** phenol-formaldehyde resin, condensation, quinone methide, mechanism

## Abstract

The mechanisms for the base-catalyzed condensation reactions in phenol-formaldehyde resin synthesis were investigated by using the density functional theory method. The structures of the intermediates and transition states, as well as the potential energy barriers of the involved reactions, were obtained. The hypothesis of quinine methide (QM) formation was theoretically confirmed. Two mechanisms were identified for QM formation, namely E1cb (elimination unimolecular conjugate base) and water-aided intra-molecular water elimination. The latter is energetically more favorable and is proposed for the first time in this work. Based on the QM mechanism, the condensation should be a unimolecular reaction because the following condensation between an ionized species (dissociated phenol or hydroxymethylphenol) with QM is much faster. The previously proposed S_N_2 condensation mechanism was found to be not competitive over the QM mechanism due to a much higher energy barrier. The condensation reaction between neutral phenol or hydroxymethylphenol and QM was also found to be possible. The energy barrier of this reaction is close to or higher than that of QM formation. Therefore, the overall condensation reaction may appear to be bimolecular if such a reaction is incorporated. The theoretical calculations in this work rationalized the discrepant results reported in previous kinetics studies well.

## 1. Introduction

The thermosetting phenol-formaldehyde (PF) resin is one of the most common adhesives in the wood industry. Although PF resin has been studied for over a century and acceptable products can be obtained, some challenges, such as a high curing temperature and low curing speed [1,2,3], have not been addressed. To understand the chemistry of PF, the influences of the reaction conditions, including the catalysts, formaldehyde to phenol molar ratio (F/P), temperature, reactant-adding procedure etc., on the changes of the molecular composites of the resin have been investigated and discussed [3,4,5,6,7,8,9], but the experimental results have not been well rationalized due to the complexity of the PF chemistry and difficulties in having insight into the involved reactions, especially the microscopic mechanisms. Therefore, the theory of PF resin synthesis has been developed slowly during the past decades.

The kinetics and mechanisms of the individual reactions in the PF system are apparently the key issues and also the foundation for understanding the chemistry of PF. However, by surveying the literature from the middle of the last century to recent years, discrepant results for the kinetics and mechanisms of the base-catalyzed condensation reactions between hydroxymethylphenols (HMPs) have been reported [9,10,11,12,13,14,15,16,17,18,19]. In some earlier studies, the condensations of HMPs were reported to be first-order [9,10,11], pseudo-first-order or second-order [12,13]. These reports implied or clearly pointed out that the condensations occurred in an S_N_2 (bimolecular nucleophilic substitution)-type mechanism. Namely, the reactions occurred between undissociated and dissociated (ionized) HMPs, dissociated and dissociated HMPs, and undissociated and undissociated HMPs. Later in 1983, Jones observed the self-condensation of trihydroxymethylphenol (THMP) to be first-order and proposed a quinone methide (QM) intermediate hypothesis to explain the kinetics [14]. In 2001, Higuchi et al. confirmed that the self-condensation of 2-hydroxymethylphenol (2-HMP) is kinetically of genuine first-order and the QM hypothesis seems to hold in such a reaction [15]. However, in their continuous studies on 4-hydroxymethylphenol (4-HMP) [16], 2,4-dihydroxymethylphenol (2,4-DHMP) [17] and 2,4,6-trihydroxymethylphenol (2,4,6-THMP) [18], the condensations appeared to be of fractional order, ranging from 1.1~2.0. Further, the reaction order varied with the change of the NaOH/HMP molar ratio and with the change of the HMP concentration. Based on these results, Higuchi et al. implied that, beside the unimolecular mechanism which is represented by the QM hypothesis, bimolecular reactions such as S_N_2 may also contribute to the reaction [16,17,18]. A very recent study [19] also suggested that both the QM and S_N_2 mechanisms work in the condensations. Obviously, the very fundamental issue in PF resin chemistry has not been addressed.

The discrepant conclusions drawn from the kinetics experiments are mainly due to the complex nature of the PF reaction system. On the other hand, the reactive intermediates are generally short lived and they cannot be directly observed or monitored by experimental methods. Fortunately, theoretical calculations on the computer based on developed quantum chemistry theory allow us to investigate each individual reaction and can provide us with a clear picture of how the reactants evolve to intermediates and finally to products.

In this study, detailed theoretical calculations were performed on the model base-catalyzed reaction between phenol and hydroxymethylphenol to clarify the key issues that have not been addressed. First, how is the quinine methide intermediate formed? How is it reactive in condensation reactions? Second, is the S_N_2 mechanism that was proposed previously also possible? If yes, which one is more favorable? Finally, are there any other new mechanisms?

The reaction pattern and mechanisms identified for phenol in this study may be extended to other phenolic compounds, such as resorsinol and tannin [20], because they have a similar phenolic functional group, although the reactivity and molecular weight of these compounds are different from phenol.

## 2. Theoretical Calculations

All the structures of the stationary points on the reaction potential energy surfaces (PES), including reactants, intermediates, transition states and products, were fully optimized by using the density functional theory [21,22] (DFT) method B3LYP [23,24,25] with the standard basis set 6-31++G** [26,27,28,29]. The harmonic vibrational frequencies were calculated to characterize the nature of the stationary point as a local minimum or first-order saddle point (transition state) on the PES. The zero-point vibrational energies (ZPVE) were used to correct the relative energies. Each transition state (TS) was identified as it has a unique imaginary frequency. Intrinsic reaction coordinate (IRC) analysis was also performed to confirm the connectivity between a TS and a local minimum. For all the calculations, the self-consistent reaction field (SCRF) method was employed with the polarizable continuum model (PCM) [30,31,32] by defining water as the solvent to simulate the implicit solvent effects (Water: ε = 78.3553). All the calculations were carried out with the GAUSSIAN 03 program package [33].

The relative energies in the potential energy profiles shown in this paper were obtained by the following formulas:

*ΔE*(*TS*) = [(*E*(*TS*) + *ZPVE*(*TS*)] − [*E*(*R*) + *ZPVE*(*R*)]

*ΔE*(*P*) = [(*E*(*P*) + *ZPVE*(*P*)] − [*E*(*R*) + *ZPVE*(*R*)]

Here, *E*(*TS*) is the total electron energy of a TS, while *E*(*R*) represents the total electron energy of a local minimum which is on the reactant direction (left side) of a TS. Thus, *ΔE*(*TS*) represents the energy barrier of a TS relative to the left local minimum on the PES. Similarly, *E*(*P*) is the total energy of electrons of a local minimum which is on the product direction (right side) of a TS, and *ΔE*(*P*) corresponds to the relative energy of a right local minimum with respect to a left local minimum. Note that the left local minimum can be initial reactants or reactant-like intermediates, and the right local minimum can be reaction products or product-like intermediates. It depends on what target reaction is being investigated.

## 3. Results and Discussion

**Figure 1:**
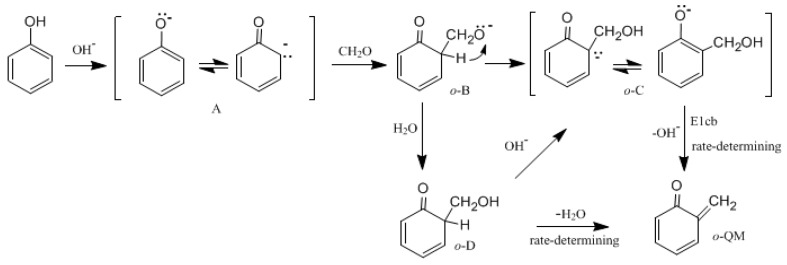
The mechanisms for quinine methide formation.

**Figure 2:**
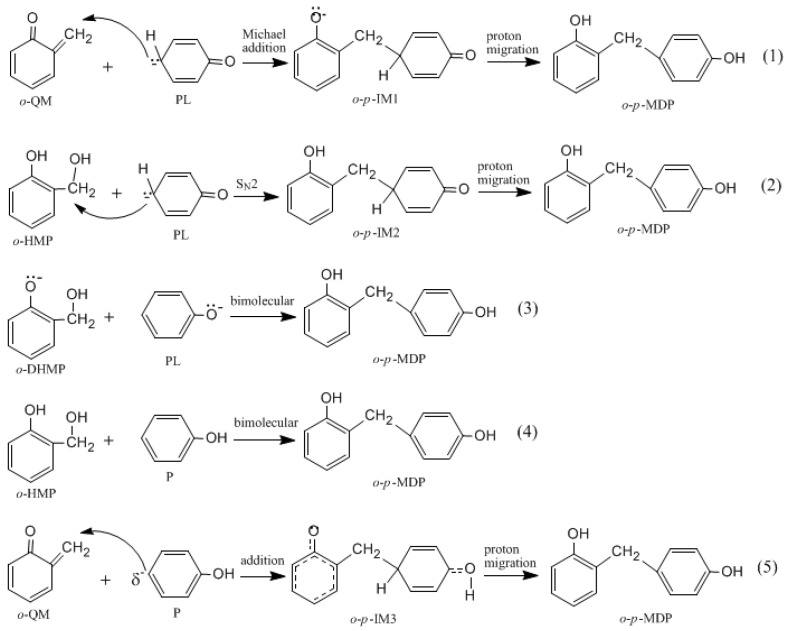
Possible mechanisms for condensation reactions (IM stands for intermediate).

Figure 1 shows the possible mechanisms for the formation of the *ortho*-quinone methide (*o*-QM) intermediate. Figure 2 shows the possible mechanisms for the condensation reactions. The first pathway leading to *o*-QM is A → *o*-B → *o*-C → *o*-QM. Another pathway is A → *o*-B → *o*-D → *o*-C → *o*-QM. Both the pathways must pass the intermediate *o*-C. Via the E1cb loss of OH^−^, the *o*-QM can be formed and this is the rate-determining step. Such a mechanism is proposed in some literature [15,16,17,18,19]. However, we considered another mechanism where intra-molecular water elimination from the intermediate *o*-D also leads to *o*-QM. The para-quinone methide (*p*-QM) can also be formed via similar mechanisms. Note that a water-elimination mechanism was also proposed by Higuchi et al. [15]. However, the initial species was thought to be a neutral HMP. We performed calculations on this mechanism, but it was not identified.

**Figure 3:**
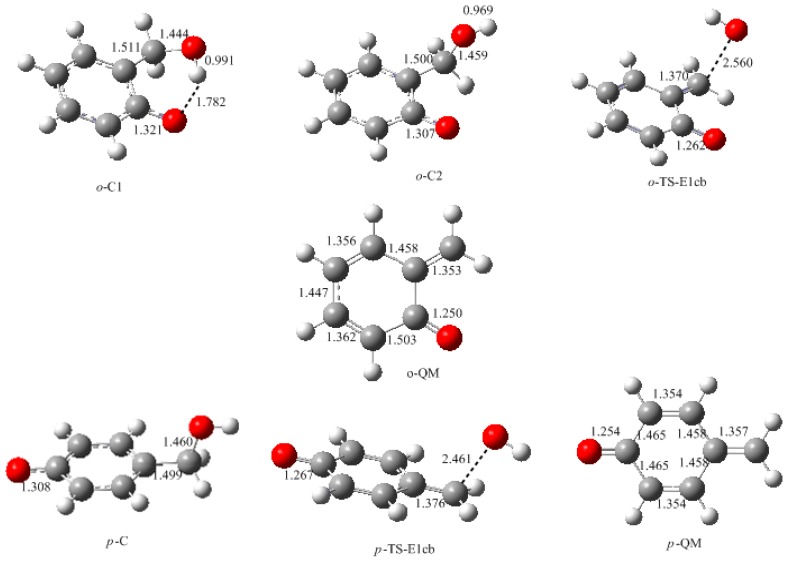
The calculated structures of the intermediates and transition states for the E1cb mechanism (selected bond lengths in Å, oxygen in red, carbon in grey and hydrogen in white).

**Figure 4:**
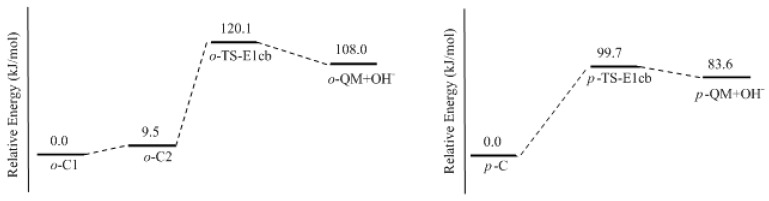
Potential energy profiles for the E1cb formation of *ortho*- and *para*-quinone methide.

In this work, the rate-determining step of E1cb and water elimination was calculated and the obtained structures are shown in Figure 3. The calculated potential energy profiles are given in Figure 4. The *o*-C1 and *o*-C2 are two structures of intermediate C, and the former is 9.5 kJ/mol more stable than the latter because a hydrogen bond (1.782 Å) can be formed between the *o*-hydroxymethyl group and the carbonyl oxygen. Via the transition state *o*-TS-E1cb, the OH^−^ can be eliminated and produce *o*-QM. The potential energy barrier of this step was calculated be 120.1 kJ/mol. For the formation of *p*-QM, this barrier was lowered to 99.7 kJ/mol. Note that *o*-QM and *p*-QM are isomers and our calculations suggest that the latter is more stable by 24.4 kJ/mol.

**Figure 5:**
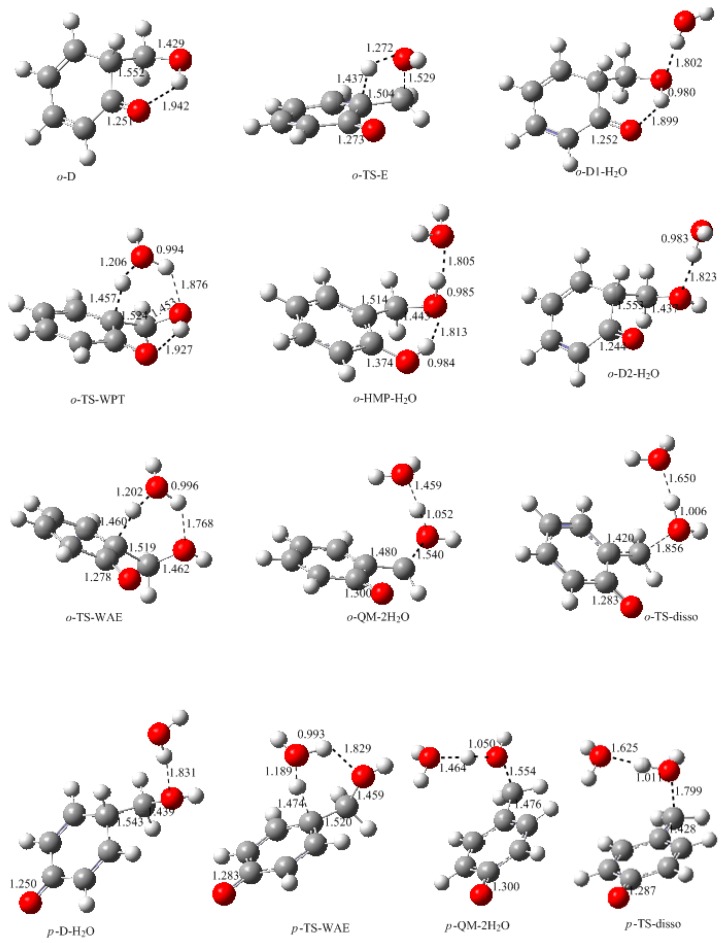
The calculated structures of the intermediates and transition states for the *ortho*- and *para*-quinone methide formation via water-aided elimination mechanism (selected bond lengths in Å, oxygen in red, carbon in grey and hydrogen in white).

**Figure 6:**
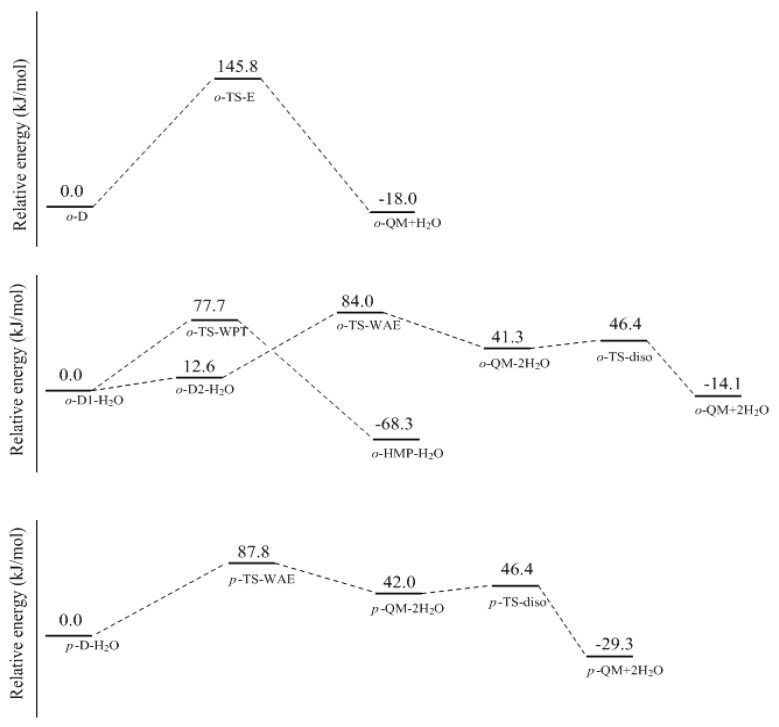
The potential energy profiles for the formation of *ortho*- and *para*-quinone methide via water-aided elimination mechanism.

However, the formation of QM will not definitely experience intermediate *o*-C or *p*-C if the initial reactants are phenol and formaldehyde. An alternative mechanism that works via water elimination from *o*-D or *p*-D is also possible. The calculated results for this mechanism are shown in Figure 5 and Figure 6. From the *ortho*-position intermediate *o*-D in which an intra-molecular bond is also formed, a water molecule can be eliminated via the four-member transition state TS-E, directly leading to *o*-QM. The energy barrier of this reaction was calculated to be 145.8 kJ/mol. Obviously, such a high barrier is not consistent with the kinetics measurement. However, in aqueous solution, the water molecule may play the role of catalyst. Therefore, we put a water molecule in the theoretical model to simulate the catalytic effect. The *o*-D1-H_2_O is the obtained intermediate complex formed through the intermolecular hydrogen bonding interaction between *o*-D1 and H_2_O. Based on this complex, the six-member water-mediated proton transfer transition state *o*-TS-WPT was identified. The vibration mode of the imaginary frequency clearly showed the proton transfer from the carbon to the water oxygen, and from the water to the hydroxyl oxygen. It is much like the expected transition state. However, IRC calculation demonstrated that once a proton shifted to the hydroxyl oxygen, another proton on the hydroxyl group simultaneously moved to the carbonyl oxygen due to the strong intra-molecular hydrogen bonding effect, leading to *o*-HMP-H_2_O which can be viewed as a complex of *o*-HMP and H_2_O. Therefore, the *o*-TS-WPT does not correspond to the water elimination and formation of *o*-QM, but corresponds to the simultaneous transfer of three protons and the formation of *o*-HMP. Despite that, in contrast to TS-E, the water transfer energy barrier was significantly lowered to 77.7 kJ/mol.

Obviously, the proton transfer from the hydroxyl group to the carbonyl group is due to the strong intra-molecular hydrogen bonding effect. If this effect is destroyed, water elimination may occur. By rotating the hydroxyl group and canceling the hydrogen bonding effect, the complex *o*-D2-H_2_O, which is 12.6 kJ/mol less stable than *o*-D1-H_2_O, was identified. After this complex, the water-aided elimination transition state *o*-TS-WAE was located. The IRC calculation showed that the proton on the carbon transferred to the hydroxyl group with the mediation of water, tending to eliminate two water molecules. Thus, *o*-QM-2H_2_O was the resulting intermediate. Further calculations confirmed that two water molecules can be eliminated via the dissociation transition state *o*-TS-disso, resulting in the formation of *o*-QM. As shown in Figure 6, the transition state *o*-TS-WAE represents the rate-determining step, and the energy barrier relative to *o*-D1-H_2_O was predicted to be 84.0 kJ/mol, which is significantly lower than the barrier of TS-E, clearly indicating the catalytic effect of water. Compared with the E1cb mechanism, this mechanism is also much more favorable. However, due to the intra-molecular hydrogen bonding effect, a large portion of the complex formed between *o*-D1 and H_2_O may exist mainly in the form of *o*-D1-H_2_O, instead of *o*-D2-H_2_O. As revealed above, the former evolved to *o*-HMP, not *o*-QM. Therefore, the inhibitory effect of hydrogen bonding on the formation of *o*-QM works here. The *para*-position QM (*p*-QM) is not kinetically more favorable than *o*-QM, although its formation is not influenced by the hydrogen bonding effect. As shown in Figure 5 and Figure 6, the *p*-TS-WAE was the identified transition state for the *para*-position water-aided elimination mechanism. The energy barrier represented by this transition state was 87.8 kJ/mol, slightly higher than that of *o*-TS-WAE. However, the addition between phenol and formaldehyde at the *para*-position is generally faster than the addition at the *ortho*-position. Therefore, the *p*-QM may have a higher concentration in the solution and the following condensation occurs mainly between the free *ortho*-postion of the aromatic ring and *p*-QM, leading to the *o*-*p* condensation structure. This can rationalize the experimentally observed result that the *o*-*p* condensation structure was predominant.

In the kinetics study of Higuchi et al. [15,16], the starting reactant was 2-hydroxymethylphenol (2-HMP) or 4-hydroxymethylphenol (4-HMP) and they estimated the activation energy barriers for the formation of *o*-QM and *p*-QM as 103 ± 5 and 78 ± 5 kJ/mol. This seems to agree with our calculations for the E1cb mechanism in which the *o*-QM is energetically less favorable. However, *o*-QM or *p*-QM may not be directly produced via the E1cb mechanism, because the *o*-C or *p*-C intermediate, which can be viewed as dissociated HMP, can evolve to intermediate *o*-D or *p*-D. As discussed above, *o*-TS-WAE and *p*-TS-WAE have a close potential energy barrier of 84.0 and 87.0 kJ/mol, respectively, but due to the intra-molecular hydrogen bonding effect, a portion of *o*-D-H_2_O will evolve to *o*-HMP instead of *o*-QM. As a result, a lower reaction rate and higher activation energy will be measured. This is in agreement with the conjecture of Higuchi et al. that the hydrogen bonding in 2-HMP has an inhibitory effect on *o*-QM formation.

**Figure 7:**
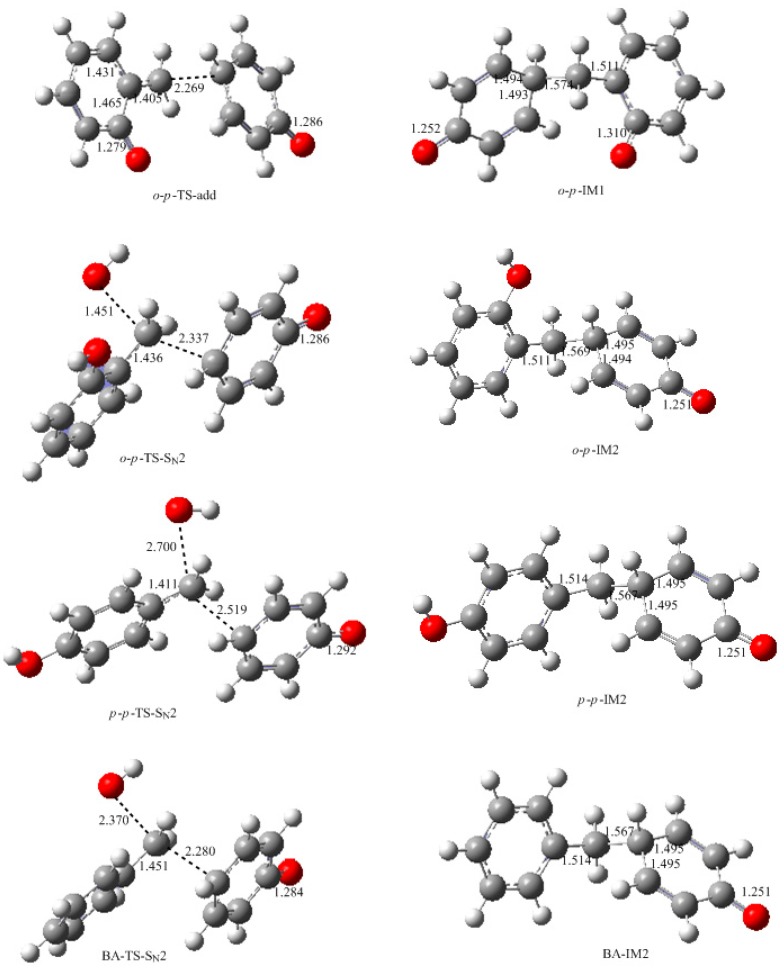
The calculated structures of the intermediates and transition states for the condensation reactions (selected bond lengths in Å, oxygen in red, carbon in grey and hydrogen in white).

**Figure 8:**
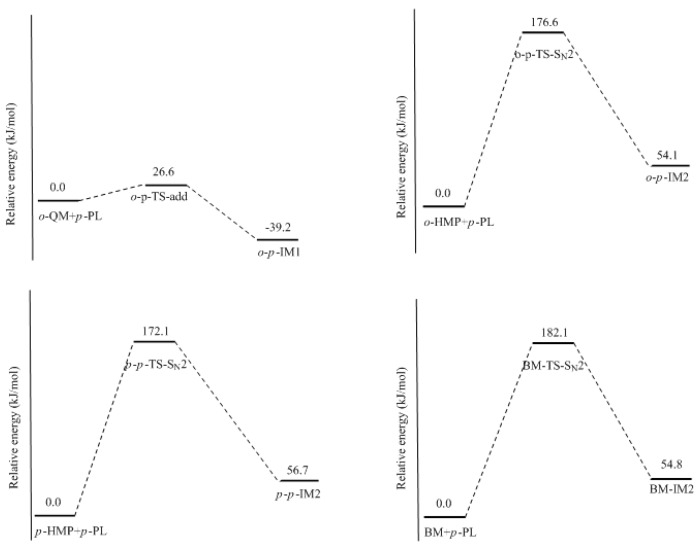
The potential energy profiles of the condensation reactions.

It was assumed in the literature that the condensation reaction of a phenolate ion (PL) or dissociated HMP with QM was much faster than the formation of QM [14,15,16,17,18,19]. To confirm this, we also performed calculations on the reaction (1) in Figure 2. As shown in Figure 7, an addition-like transition state, *o*-*p*-TS-add, was located. In fact, this is the typical Michael addition. The potential energy profile in Figure 8 shows this step has a small barrier of 26.6 kJ/mol, indicating this reaction is indeed much faster than QM formation. Proton migrations after the intermediate *o*-*p*-IM1, leading to *o*-*p*-methylenediphenol (*o*-*p*-MDP), were also assumed to be fast reactions. Therefore, further theoretical calculations were not carried out.

The S_N_2 mechanism shown in Figure 2 as reaction (2) was also suggested by some early reports [9,10,11,12,13]. However, in the study of Higuchi et al. on the self-condensation of 2-HMP (*o*-HMP) [15], the authors concluded that the reaction is of genuine first-order and any bimolecular mechanism should be ruled out. To clarify the competitive relationship of the QM and S_N_2 mechanisms, we performed calculations on the S_N_2 reaction. In Figure 7, the S_N_2 transition state *o*-*p*-TS-S_N_2 demonstrates that the collision between the *o*-HMP and the *para*-position of dissociated phenol (phenolate ion) can lead to the removal of OH^−^, forming the intermediate *o*-*p*-IM2. The energy barrier of this reaction was predicted to be 172.1 kJ/mol, which is about 70 and 90 kJ/mol higher than that of the two QM mechanisms, respectively. Obviously, such a mechanism is not competitive over the QM mechanism. This results rationalized the kinetics results of Higuchi et al. on the self-condensation of 2-HMP. However, in their study on the self-condensation of 4-HMP (*p*-HMP) [16], the reaction order was found to be 1.3 and they thought a bimolecular reaction may be involved. Subsequently, the S_N_2 mechanism was proposed again. To confirm whether 4-HMP is more reactive than 2-HMP in the S_N_2 reaction, the *para*-*para* reaction was also calculated and the transition state is shown in Figure 7 as *p*-*p*-TS-S_N_2. The barrier of it was calculated to be 172.1 kJ/mol. Therefore, the S_N_2 mechanism for 4-HMP is also not competitive over the QM mechanism. To rationalize their results, they thought the bimolecular reactions between two dissociated 4-HMPs or between two undissociated 4-HMPs, which are shown in Figure 2 as reaction (3) and (4), may be possible. However, they also doubted the rationality of the S_N_2 reaction mechanism in these two types of reactions, and finally they concluded that some unknown mechanisms may be at work. We also performed calculations on reaction (3) and (4), but they were not identified.

To further confirm the role of the S_N_2 mechanism, we have did a simple experiment on a model reaction where the phenol was reacted with benzyl alcohol under the conditions of pH = 10, 90 °C. The ^13^C NMR determination was performed for analyzing the reaction products. Benzyl alcohol (BA) is structurally similar to HMP in the aspect of the aromatic hydroxymethyl group but the QM intermediate cannot be formed from it. The S_N_2 mechanism works if the condensed structure can be formed. However, the ^13^C NMR spectrum did not indicate any condensation structure or other products within 180 min. This is why we did not show the experimental results here. To theoretically compare the reactivity of BA with HMP, we calculated the S_N_2 reaction between BA and the phenolate ion. The structure of the transition state and the reaction potential energy profile are also given in Figure 7 and Figure 8, respectively. The energy barrier for this reaction was calculated to be 182.1 kJ/mol, close to that of the reaction between the HMP and phenolate ion. This result suggests that the S_N_2 reaction is very unlikely to occur. Note that the nulceophile in this study is always an unsubstituted phenolate ion, not dissociated (ionized) HMP. Theoretically, dissociated HMP should be a weaker nucleophile due to the electron-withdrawing effect of the hydroxymethyl group. Therefore, the S_N_2 reaction between dissociated HMP and undissociated HMP is more impossible.

**Figure 9:**
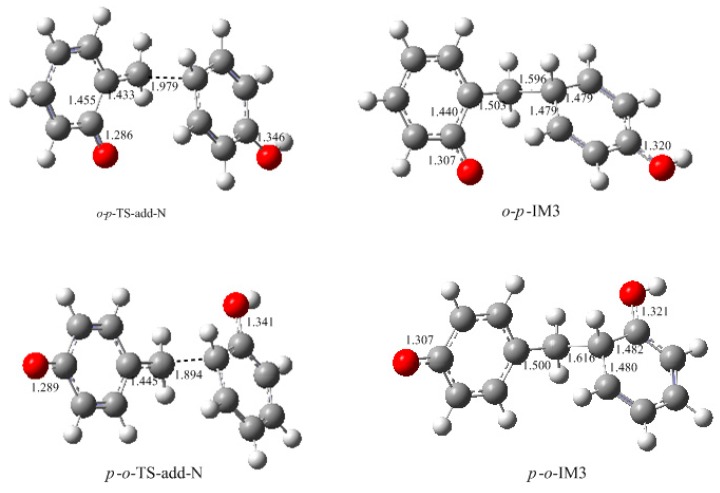
The structures of intermediates and transition states for the condensations between *ortho*-, *para*-quinone methide and the neutral phenol (selected bond lengths in Å, oxygen in red, carbon in grey and hydrogen in white).

**Figure 10:**
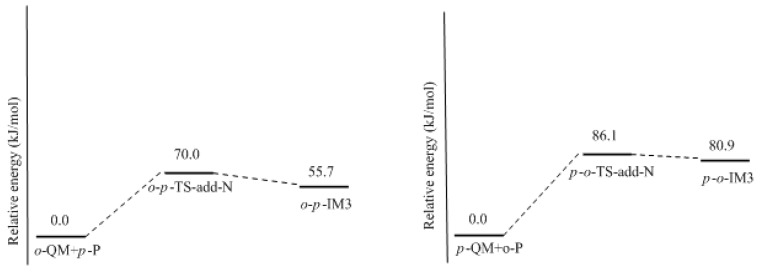
The potential energy profiles of the condensations between *ortho*-, *para*-quinone methide and the neutral phenol.

How to explain the experimentally observed bimolecular feature? We considered another possible mechanism. That is the reaction between QM and a neutral phenol or HMP. In such a reaction, the neutral phenol or HMP is a weaker nucleophile than the dissociated species. Moreover, the phenol is a weak acid, and even with the presence of a base such as NaOH, a portion of the phenol or HMP is still in neutral form. Higuchi et al. found that the bimolecular reaction rate varied with the change of the NaOH/4-HMP molar ratio [16]. Especially when the molar ratio was higher than 0.5, the reaction rate began to decrease. This implies that some kind of reaction that involves the neutral species may occur. Based on this context, we proposed a new mechanism where the QM intermediate reacts with the neutral phenol. The results are given in Figure 9 and Figure 10. The energy barrier for the reaction between *o*-QM and the *p*-position of the neutral phenol was 70.6 kJ/mol, which is still lower than the barrier of *o*-TS-WAE. The rate-determining step was still the formation of *o*-QM. The energy barrier for the reaction between *p*-QM and the *ortho*-position of neutral phenol was calculated to be 86.1 kJ/mol, which is closer to the barrier of 87.8 kJ/mol for *p*-QM formation. When we alternated the neutral phenol to 4-HMP, this barrier was calculated to be 87.6 kJ/mol (not shown in Figure 10 due to the similarity), suggesting the substituent effect on this reaction is very weak. It is possible that the theoretical calculation underestimated the barrier of this reaction to some extent due to the difficulties in simulating the real solution for the theoretical solvent model. If this reaction becomes the rate-determining step, the overall reaction will appear as a bimolecular feature. To say the least, even the barrier of the addition step is really slightly lower, they are very close indeed. Therefore, it is not safe to experimentally judge that the reaction is unimolecular.

## 4. Conclusions

The mechanisms of the base-catalyzed condensation reactions in phenol-formaldehyde resin synthesis were investigated by using the density functional theory method and the main conclusions have been drawn as follows:

(1) The quinine methide (QM) hypothesis was confirmed by theoretical calculations. Besides the E1cb mechanism, a water-catalyzed intra-molecular elimination mechanism was identified. This new mechanism is energetically more favorable.

(2) The condensation between the phenolate ion and QM is much faster than QM formation, agreeing with the experimentally observed unimolecular feature of the overall reaction.

(3) The previously proposed S_N_2 mechanism for the condensation reaction was ruled out due to the much higher energy barrier compared to the QM mechanism.

(4) The condensation of a neutral phenol or hydroxymethylphenol with QM is also possible. The calculated barrier of such a reaction is very close to that of QM formation. Incorporation of neutral species in condensations may make the reaction kinetically bear bimolecular features.
